# Efficacy on gait and posture control after botulinum toxin A injection for lower-limb spasticity treatment after stroke: A randomized controlled trial

**DOI:** 10.3389/fnins.2022.1107688

**Published:** 2023-01-16

**Authors:** Hui-xian Yu, Si-hao Liu, Zhao-xia Wang, Chang-bin Liu, Pei Dai, Da-wei Zang

**Affiliations:** Department of Rehabilitation Medicine, Beijing Tiantan Hospital, Capital Medical University, Beijing, China

**Keywords:** stroke, botulinum toxin A, gait, spasticity, posture control

## Abstract

**Objectives:**

To observe the efficacy of botulinum toxin type A (BoNT-A) for the spasticity of the lower-limb post-stroke on gait and posture control.

**Methods:**

A total of 46 patients with hemiplegia gait were randomly divided into the experimental group (23 patients) and the control group (23 patients). In patients in the experimental group received injections of BoNT-A by electrical stimulation-guided. At the same time, patients of the two groups received routine physical therapy. Gait analysis, plantar pressure analysis, lower-limb Fugl–Meyer assessment (L-FMA), 10 meter walking test (10MWT), timed “Up and Go” test (TUGT), and modified Ashworth Scale assess (MAS) of the lower limbs were performed at 0, 1, 4, and 12 weeks after treatment.

**Results:**

At 1, 4, and 12 weeks after treatment, the L-FMA, stride length, speed, and TUGT significantly improved than 0 week in both groups. The L-FMA and peak of forefoot pressure, and MAS results in the experimental group were better than those in the control group at 4 and 12 weeks. The TUGT, speed, and stride length in experimental group was significantly shortened than that in control group at 1, 4, and 12 weeks.

**Conclusion:**

Botulinum toxin type A injection can improve motor functions of the lower limb, gait, spasticity, forefoot pressure, and posture control of patients after stroke.

## Introduction

Stroke is one of the leading causes of death and adult disability globally (Wang, 2022). A total of 75% of patients who sustain a stroke have limitations in walking (Abilleira et al., 2005), and the most common pattern of walking impairment post-stroke is hemiparetic gait. Patients with post-stroke hemiparesis may experience classic changes in spatiotemporal, kinematic, and kinetic parameters. A hemiplegic gait is often described as insufficient knee flexion or knee hyperextension, hip lifting, external rotation, ankle varus, and plantarflexion of the affected side limb (Sheffler and Chae, 2015; Nikamp et al., 2019). Most of this gait pattern is caused by spasticity. The spasticity after central nervous system injury is the main cause of this typical abnormal pattern. Spasticity is a common complication in upper motor neuron syndrome after neurological diseases, which is characterized clinically by a velocity-dependent increase in muscle tone and the stretch reflex. In the single hemiplegic side limb-supporting phase, the foot is in the abnormal position. This equinovarus foot posture affects gait, standing, and weight transfer (Tao et al., 2015). Furthermore, spasticity of the lower limb can impair the function of daily activities and increase the risk of falls (Thibaut et al., 2013).

**FIGURE 1:**
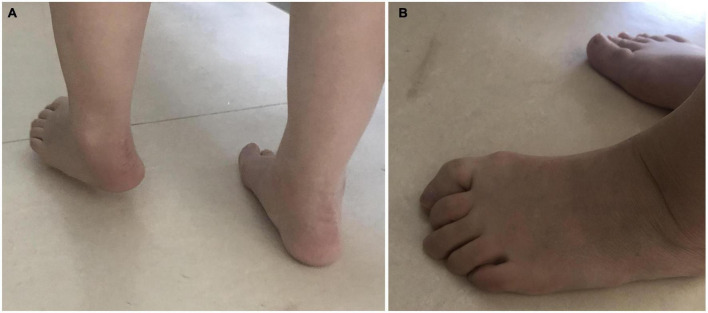
Common patterns of varus and toe flexion after stroke. **(A)** Foot drop; the heel can not land first replaced by the anterolateral edge of the foot. **(B)** In the weight-bearing phase, the spasm of the flexion muscle of the toes increases with the increase of the weight of the affected limb, the arch of the feet cannot support the weight stably. In the off-ground, the toes cannot be lifted off the ground by dorsiflexion.

The spasticity should be treated in time, triceps spasm in calves causes forefoot landing and knee hyperextension (Sankaranarayan et al., 2016). Spasms of the posterior tibialis muscle and flexor digitorum longus (FDL) muscle can cause strephenopodia and toe flexion (like Figure 1). This abnormal movement pattern leads to a decrease in walking speed and falls (Paula et al., 2019a).

Botulinum toxin type A injections are effective in treatments available for spasticity (Baker and Pereira, 2016). BoNT-A is used to reduce the excessive activity of the focal muscles of upper motor neuron injured syndrome (Pandyan et al., 2005). BoNT-A can induce weakness, relax the target muscles temporarily, and make them easier to be extended. This can alleviate neurogenic and biomechanical factors that contribute to the spasticity.

Studies have shown that Botox injection can significantly reduce lower limb muscle tone (Kaňovsk et al., 2021), improve walking speed (Marciniak et al., 2019), and improve ankle motion (Marciniak et al., 2019). However, there is insufficient evidence to support the improvement of lower limb gait and postural control. There are fewer data reporting the efficacy of BoNT in well-designed clinical trials in adults with lower-limb spasticity (Santamato et al., 2019). In this study, we used a plantar pressure system and performed gait analysis to evaluate the effect of BoNT-A injection on the lower limb, gait, and posture control function.

## Materials and methods

### Study design

The study was performed as a randomized controlled trial with single-blinded assessments. The assessors and statisticians were blinded to the group assignment. That was approved by the Ethics Committee of Beijing Tiantan Hospital, Capital Medical University (KY2020-069-01). The registration number of this clinical trial was ChiCTR2000037479.

### Study participants

A total of 46 patients with hemiplegia gait after stroke localized to the corticospinal tract were included in the Department of Rehabilitation Medicine of Beijing Tiantan Hospital from 1st January, 2021 to 31st July, 2022. This study used nets sample size calculator. The patients were randomly divided into treatment and control groups using random number table^1^ (23 patients in each group). Randomization was performed by one doctor who was not involved in other work of this study. The inclusion criteria were as follows: (1) first-ever stroke; (2) age of 35–65 years; (3) ≤6 months post-stroke; (4) they had slight spasticity at resting state of the triceps surae as defined by a score of 1−1+ on the MAS or ankle clonus (+); (5) at Brunnstrom recovery stage III–IV; (6) walking with hyperextension or foot drop or toe flexion deformity. (7) no obvious effect after oral anti-spasticity medications for at least 1 month. (8) Mini-mental state examination (MMSE) score > 25. The exclusion criteria were as follows: (1) other cerebrovascular diseases; (2) sensory impairment; (3) other osteoarticular system diseases; (4) history of BoNT injection; (5) severe allergic constitution.

All participants signed informed consent.

### Study protocol

#### Intervention

In the control group, patients received routine rehabilitation treatment, including oral anti-spasticity medications and the use of ankle-foot orthotics. In this study, we did not intervene in the choice of medication, method of rehabilitation, and brace type. In the experimental group, patients received BoNT-A (Allergan) injection and routine rehabilitation treatment without oral anti-spasticity medications. The injection was administered by the same physician using electrical stimulation-guided injection. The injection dose and target sites were selected by the physician after individual assessment, referring to Chinese guidelines for the treatment of adult limb spasm with BoNT (Li et al., 2015). The main target muscles were selected mainly depending on gait patterns including quadriceps femoris (QF), gastrocnemius (GS), tibialis posterior (TP), flexor hallucis longus (FHL), FDL, FD brevis (FDB), and FH brevis (FHB) [specific operations followed table (Table 1)].

**TABLE 1:** BoNT-A injection scheme protocol.

Target muscles	Number	Dose (IU)
QF + GS + TP	3	150 + 150 + 70
GS + TP + FDL + FHL	8	150 + 70 + 70 + 50
GS + TP + FDL + FHL + FDB + FHB	7	150 + 70 + 70 + 50 + 20 + 10
GS + TP	3	150 + 70

QF, quadriceps femoris; GS, gastrocnemius; TP, tibialis posterior; FHL, flexor hallucis longus; FDL, flexor digitorum longus; FDB, flexor digitorum brevis; FHB, flexor hallucis brevis.

#### Outcome measures

All patients were evaluated using a modified Ashworth Scale assess (MAS), Fugl–Meyer assessment (FMA) of the lower limbs, gait analysis, dynamic plantar pressure analysis, and timed “Up and Go” test (TUGT) at 0, 1, 4, and 12 weeks. These assessments were performed by another physician.

Modified Ashworth Scale assess was used to evaluate the spasticity of the GS muscle, and the score ranged from 0 to 5 grades (instead of 0, 1, 1+, 2, 3, and 4).

The motor function of the lower limb was assessed using the lower-limb FMA (L-FMA) motor score (from 0 to 34 grades). The walk and balance functions were assessed using a gait analysis and plantar pressure analysis system (zebris FDM 1.12).

#### 10 meter walking test

Patients were asked to walk 12 m forward in a state of natural speed, assessor recorded the time they spent between 1 and 11 m (Yeung et al., 2018).

#### TUGT

The patients were required to sit on the chair after which the researcher began to record with a stopwatch from the moment the patient got up, walked for 3 m, turned around the cone, and returned to sit down (Dong et al., 2021).

Zebris gait analysis and plantar pressure measurement system (zebris FDM 1.12) was used in this study. The patients were required to take off the shoes on the platform. The participant was asked to release the railings, turn on the evaluation device, and then stop the device after walking for the 30 s. The device mainly recorded the data of both lower limbs while walking, including the peak plantar pressure (N/cm^2^) of the forefoot and heel, and the gait parameters.

Follow-up was performed at 1, 4, and 12 weeks after injection.

## Statistical analysis

Statistical analyses were performed using GraphPad Prism 8.0 (GraphPad Software, Inc., San Diego, CA, USA). All data sets were tested for normality. Descriptive summary statistics for differences between the two groups of baseline data are presented using the Kruskal–Wallis test. Continuous variables are expressed as the mean ± standard deviation. Differences within the groups before and after treatment were analyzed using one-way analysis of variance (ANOVA). Differences between the two groups were analyzed using unpaired *T* test. A *p*-value of < 0.05 was considered statistically significant.

## Results

**FIGURE 2:**
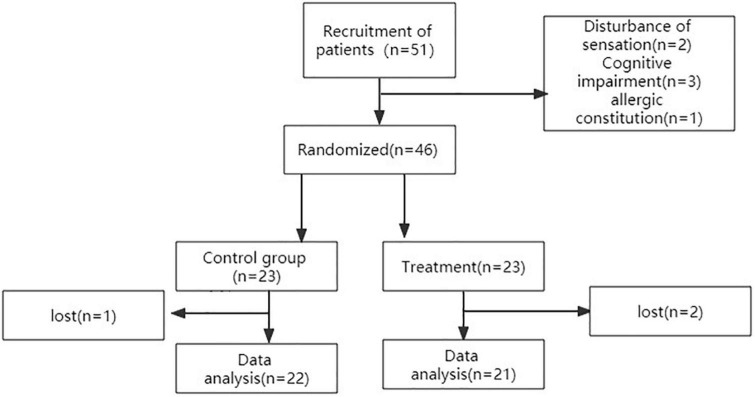
The flow diagram of the study.

During the follow-up, three patients did not complete the study (two from the experimental group and one from the control group). Among these, one from the experimental group had a second stroke during follow-up, whereas the other from the experimental group patient could not reach the hospital on time to complete the evaluation. The one from the control group patient had a hip fracture because of a fall. Thus, 43 patients completed the follow-up (21 from the experimental group and 22 from the control group). No side effects were reported. The flow diagram for enrollment and outcomes like Figure 2.

The statistical results can be shown in Table 2. In the control group, the scores of MAS were increased for the three patients at 1 and 4 weeks. A total of 8 patients showed no changes in MAS, and 11 patients had improved muscular tone. In the experimental group, all the scores of MAS decreased after injection and until 12 weeks.

**TABLE 2:** Baseline characteristics of active and sham groups.

Group	Age (years)	Gender (*n*)	Hemiplegia side (*n*)	Time (*m*)	Disorders (*n*)	Focal site
Control group	54.37 ± 9.78	F 8	L 12	3.4 ± 0.16	CH 7	BG 16 BS 6
		M 14	R 10		CI 15	
Experimental group	56.47 ± 6.82	F 5	L 13	4.2 ± 0.09	CH 4	BG 14 BS 7
		M16	R 8		CI 17	

CI, cerebral Infarct; CH, cerebral hemorrhage; BG, basal ganglia; BS, brain stem.

**FIGURE 3:**
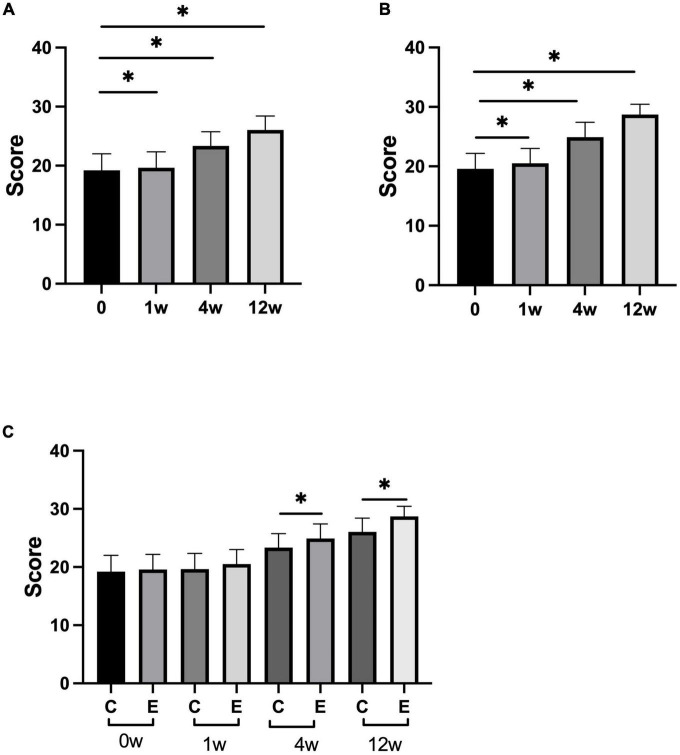
Changes in lower-limb Fugl–Meyer assessment (L-FMA) scores. **(A)** Scores of L-FMA at 1, 4, 12 weeks compared with that at 0 week in control group; **(B)** scores of L-FMA at 1, 4, 12 weeks compared with that at 0 week in experimental group; **(C)** scores of L-FMA at 0, 1, 4, and 12 weeks in experimental group compared with control group; *p < 0.05.

At 1, 4, and 12 weeks after treatment, L-FMA significantly improved compared with that before treatment in both the groups (control group: *P* = 0.000; experimental group: *P* = 0.000). Analysis between the two groups showed significant improvement in L-FMA in the experimental group compared with the control group at 4 and 12 weeks (*P* = 0.000), there was no significant difference between the two groups at 1 week (*P* = 0.0736; Figure 3).

**FIGURE 4:**
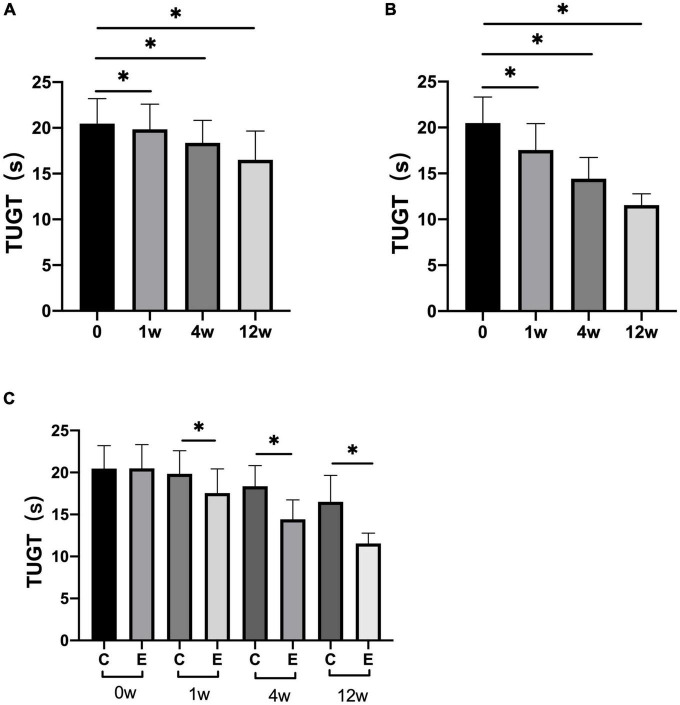
Changes in timed “Up and Go” tests (TUGTs). **(A)** TUGT at 1, 4, and 12 weeks compared with that at 0 week in control group; **(B)** TUGT at 1, 4, and 12 weeks compared with that at 0 week in experimental group; **(C)** TUGT at 0, 1, 4, and 12 weeks in experimental group compared with control group; *p < 0.05.

At 1, 4, and 12 weeks after treatment, TUGT significantly decreased compared with that before treatment in both the groups (control group: *P* = 0.000; experimental group: *P* = 0.000). Analysis between the two groups showed significant improvement in TUGT in the experimental group compared with the control group (*P* = 0.000; Figure 4).

**FIGURE 5:**
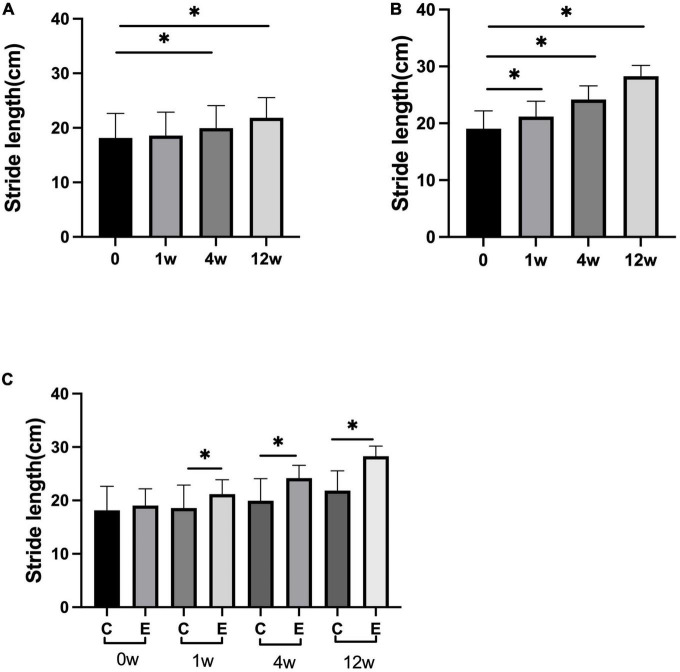
Changes in stride length (cm). **(A)** Stride length at 1, 4, and 12 weeks compared with that at 0 week in control group; **(B)** stride length at 1, 4, and 12 weeks compared with that at 0 week in experimental group; **(C)** stride length at 0, 1, 4, and 12 weeks in experimental group compared with control group; *p < 0.05.

The stride length was significantly improved compared with that before treatment in both the groups at 4 and 12 weeks after treatment (control group: 4 weeks *P* = 0.010 and 12 weeks *P* = 0.000; experimental group: *P* = 0.000). Analysis between the two groups showed significant improvement in stride length in the experimental group compared with the control group (*P* = 0.000). At 1 week, no significant difference was observed compared with that before treatment in the control group (*P* = 0.8118). In the experimental group, a significant difference was observed at 1 week compared with that before treatment (*P* = 0.000; Figure 5).

**FIGURE 6:**
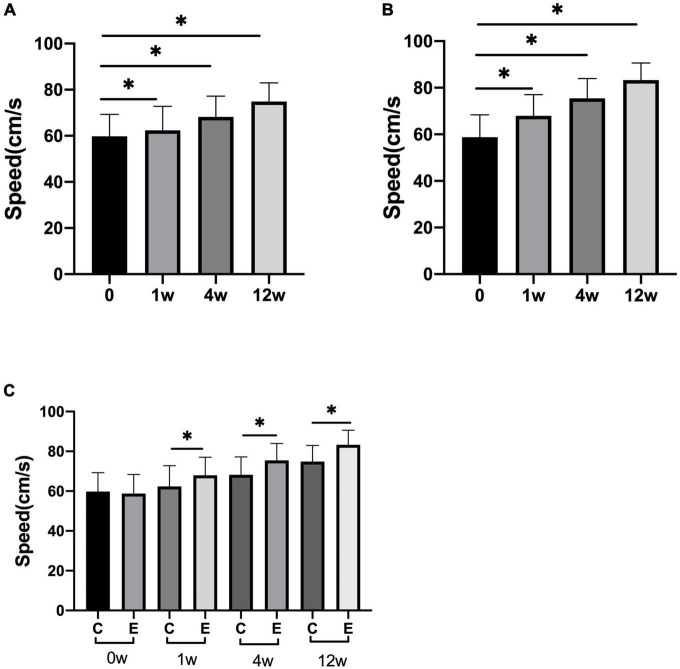
Changes in speed of 10 meter walking test (10MWT) (cm/s). **(A)** The speed at 1, 4, and 12 weeks compared with that at 0 week in control group; **(B)** speed at 1, 4, and 12 weeks compared with that at 0 week in experimental group; **(C)** speed at 0, 1, 4, and 12 weeks in experimental group compared with control group; *p < 0.05.

The speed of 10 meter walking test (10MWT) in the experimental group was higher than that of the control group (control group at 1 week *P* = 0.027, the others and experimental group *P* = 0.000). In the experimental group, the speed significantly improved at 1, 4, and 12 weeks compared with 0 week (*P* = 0.000). Analysis between the two groups showed significant improvement in speed of walking in the experimental group compared with the control group (*P* = 0.000; Figure 6).

**FIGURE 7:**
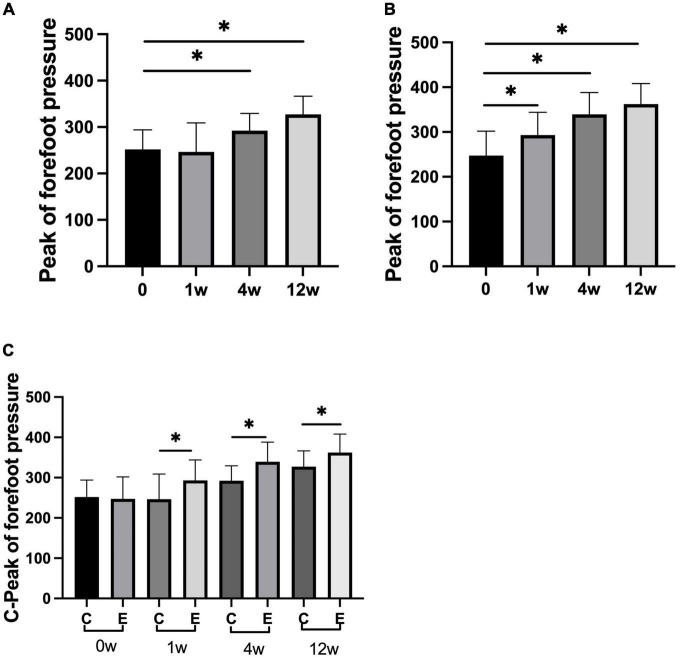
Changes in peak of forefoot pressure (*N*). **(A)** Forefoot pressure in control group at 1, 4, and 12 weeks compared with that at 0 week; **(B)** forefoot pressure at 1, 4, and 12 weeks compared with that at 0 week in experimental group; **(C)** forefoot pressure at 0, 1, 4, and 12 weeks in experimental group compared with control group; *p < 0.05.

**FIGURE 8:**
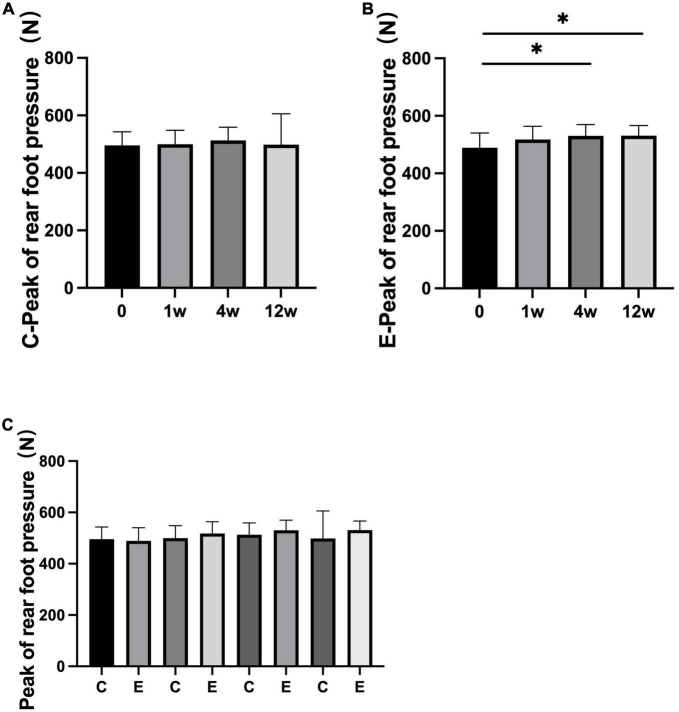
Changes in peak of rear foot pressure (*N*). **(A)** Rear foot pressure in control group at 1, 4, and 12 weeks compared with that at 0 week; **(B)** rear foot pressure at 1, 4, and 12 weeks compared with that at 0 week in experimental group; **(C)** rear foot pressure at 0, 1, 4, and 12 weeks in experimental group compared with control group; *p < 0.05.

The peak of forefoot pressure in the experimental group was significantly higher than that in the control group (*P* = 0.000). No significant change was observed after 1 week compared with that after 0 week in the control group (*P* = 0.967). At 4 and 12 weeks after treatment, forefoot pressure was significantly increased compared with that at 0 week (*P* = 0.000). In the experimental group, it significantly improved at 1, 4, and 12 weeks compared with 0 week (*P* = 0.000; Figure 7). No significant difference was observed in the peak of rear foot pressure between the two groups (0 week: control group and experimental group *P* = 0.993; 1 week: control group and experimental group *P* = 0.543; 4 weeks: control group and experimental group *P* = 0.323; and 12 weeks: control group and experimental group *P* = 0.145). The peak of rear foot pressure did not significantly change at 1, 4, or 12 weeks compared with 0 week in the control group after treatment (1 week: *P* = 0.956, 4 weeks: *P* = 0.550, and 12 weeks: *P* = 0.265). After injection, forefoot pressure significantly increased at 4 and 12 weeks compared with 0 week in the experimental group (4 weeks: *P* = 0.017 and 12 weeks: *P* = 0.035; Figure 8).

## Discussion

In this study, we studied whether a lower-limb injection of BoNT-A in the spastic muscle of patients with stroke could improve their walking ability. BoNT-A injection could significantly improve motor function of the lower limb, gait, spasticity, forefoot pressure, and posture control of patients.

A common incapacitating disease in adults is stroke (Araujo et al., 2018). Motor function is the primary treatment strategy that is most essential for the ability to perform daily activities independently for patients and families (Tyson and Kent, 2013). After a stroke, most of the abnormal movement patterns are caused by spasticity. Gracies et al. (2017) reported that BoNT-A injection for lower-limb treatment significantly reduced the MAS of the triceps calf after 4 weeks of injection, and they also proved that in the case of repeated injection, patients could have continuous improvement in muscle tone. Fietzek et al. (2014) reported that BoNT-A reduced the MAS scores over all follow-up data after 24 weeks after injection. In this study, the MAS of some patients in the control group was worsened, and some patients showed no change. Upper limb treatment for spasticity reportedly improves muscle tone after stroke; however, treatment outcome after lower limb or pes equinovarus has not been reported (Wissel et al., 2017). Therefore, the ability of BoNT-A to improve lower-limb spasticity and its benefits in treating lower-limb spasticity and spastic equinus foot cannot be ignored. In the experimental group, the MAS of all patients was better than that before injection.

Rousseaux et al. (2014) reported that after BoNT-A injection, the spasticity, passive range of motion, limb positioning, and pain were improved, but the motor function remained unchanged. Controversy exists about improvements in motor function after BoNT-A treatment, Wu et al. (2016) demonstrated the improvement of FMA, but the gait speed was not improved in a systematic review and meta-analysis. Tao et al. (2015) reported BoNT-A injection can increase a FMA at 4 and 8 weeks after treatment. In this study, rehabilitation could improve L-FMA, and the motor function was better after BoNT-A injection than in the control group.

Tao et al. (2015) also reported that BoNT-A injection can increase a patient’s stride length, speed at 4 and 8 weeks. Bensmail et al. (2021) reported that BoNT-A injection can improve functional ambulation classification scale scores after 2 weeks and improve independent walking. Gracies et al. (2015) reported that walking speed of patients improvement at 12 weeks after injection. This study showed significant improvement in speed of walking in the experimental group compared with the control group.

The most common synkinesis motion pattern of lower extremity extensor muscles, a fundamental principle of motor control, is the alternating contraction of antagonistic flexor and extensor muscles, which is required for all motor behaviors, including locomotion (Ausborn et al., 2018). Spastic equinus foot is caused by spasms of calf triceps, FDL, or FHL and reduces the lower limb stability and affects walking and hemiplegic lower limb weight bearing (Ferreira et al., 2013). Thus, the pressure of the center of gravity in the weight-bearing phase cannot be transferred forward to the medial side. Spasticity can exacerbate asymmetrical hemiplegia gait that includes equinus foot. Gait analysis after BoNT-A injection has been performed in a few studies, but analysis of plantar pressure after BoNT-A injection has not been reported. Manipulative treatment and bracing treatment are commonly used for treating foot drop (Ibuki et al., 2010; Paula et al., 2019b). Clinical observations in our study have shown that the patients were often accompanied by obvious foot varus and spasms of the toe muscles (Yu, 2021). After a stroke, treating foot varus is more difficult than treating foot drop. In a walking cycle, the lateral edge of the foot lands, and in the support phase, plantar pressure cannot sufficiently be transferred from the hindfoot to the medial side of the forefoot (Tao et al., 2015; Yu, 2021).

In this study, although stride length improved significantly in both the groups, the improvement was greater in the injection group and BoNT-A injection could increase the pressure on the forefoot more significantly than that in the control group. The gait control of patients after BoNT-A injection was better than that of patients in from the control group. BoNT-A injection can improve the extensibility of lower limb and plantar muscles, which can transfer plantar pressure from the lateral edge to the anterior side of the foot, and improve load-bearing capacity and gait stability.

Timed “Up and Go” test can be used to detect the risk of falls in stroke patients. Based on the results of this study, the improved TUGT test can record postural control ability in more detail under different motor modes (Yu et al., 2021). This study showed that rehabilitation could improve TUGT, and the motor function was better after BoNT-A injection than in the control group, which means BoNT-A injection can improve postural control more effectively and reduce the risk of falling. Few research about the plantar pressure and TUGT after injection of BoNT-A was reported, so more research is needed.

## Limitation

This study was a small sample, single center study, results, and data were not comprehensive. The design of the experiment may not be very rigorous. At the same time, due to the limited conditions, our evaluation equipment is unadvanced and lacks more objective indicators. It is hoped that a multi-center study will be carried out in future studies, and more objective indicators such as electromyography and ultrasound will be collected.

## Conclusion

Botulinum toxin type A injection can improve gait and postural control and reduce the risk of falls for patients with pes equinovarus due to triceps surae and toe muscle spasm and other patterns of lower-limb post-stroke spasticity.

## Data availability statement

The raw data supporting the conclusions of this article will be made available by the authors, without undue reservation.

## Ethics statement

The studies involving human participants were reviewed and approved by the Ethics Committee of Tiantan Hospital. The patients/participants provided their written informed consent to participate in this study.

## Author contributions

D-WZ and H-XY: conceptualization. H-XY: methodology, investigation, writing—original draft preparation, visualization, and project administration. C-BL: software. D-WZ, S-HL, and H-XY: validation. Z-XW: formal analysis and data curation. H-XY and PD: resources. H-XY and S-HL: writing—review and editing. PD: supervision. D-WZ: funding acquisition. All authors have read and agreed to the published version of the manuscript.

## Footnotes

1
http://www.calculator.net/sample-size-calculator.html
